# Medical education assessment: a brief overview of concepts in generalizability theory

**DOI:** 10.5116/ijme.5278.a850

**Published:** 2013-09-11

**Authors:** Mohsen Tavakol, Robert L. Brennan

**Affiliations:** 1 Medical Education Unit, The University of Nottingham, UK; 2 Centre for Advanced Studies in Measurement and Assessment, The University of Iowa, USA

General Medical Council (GMC) in the UK has emphasized the importance of internal consistency for students’ assessment scores in medical education.^1^ Typically Cronbach’s alpha is reported by medical educators as an index of internal consistency. Medical educators mark assessment questions and then estimate statistics that quantify the consistency (and, if possible, the accuracy and appropriateness) of the assessment scores in order to improve subsequent assessments. The basic reason for doing so is the recognition that student marks are affected by various types of errors of measurement which always exist in student marks, and which reduce the accuracy of measurement. The magnitude of measurement errors is incorporated in the concept of reliability of test scores, where reliability itself quantifies the consistency of scores over replications of a measurement procedure. Therefore, medical educators need to identify and estimate sources of measurement error in order to improve students’ assessment.Under the Classical Test Theory (CTT) model, the student’s true score is the sum of the student’s observed score and a single undifferentiated error term. Using this model, the most frequently reported estimate of reliability is Cronbach’s alpha. Almost always, however, when alpha is reported, it incorporates errors associated with sampling of items, only. Accordingly, alpha does not allow us to pinpoint, isolate, and estimate the impact of *different* sources of measurement error associated with observed student marks. An extension of CTT called “G (Generalizability) theory” enables us to differentiate the multiple, potential sources of measurement error called “facets” (sometimes called “dimensions” in experimental design literature). For example, in an OSCE exam, a student might be observed by one of a large sample of examiners, for one of a large sample of standardized patients (SPs), and for one of a large sample of cases. The facets, then, would be examiners, SPs and cases---each of which serves as a potential source of measurement error. The set of all facets constitutes the universe of admissible observations (UAO) in the terminology of G theory. As another example, suppose that for a cardiology exam, the investigator is interested in an item facet, only; in that case, there is only one facet.There is no right answer to the question of which facets, or how many facets, should be included in the UAO. It is the investigator’s responsibility to justify any decision about the inclusion of facets, and provide supporting evidence about the importance of each facet to the consistency and accuracy of the measurement procedure. G theory provides a conceptual framework and statistical machinery to help an investigator do so.For any given form of a test, there are a specified number of conditions for each facet. The (hypothetical) set of all forms similarly constructed is the called the universe of generalization (UG). For any given examinee, we can conceive of getting an average score over all such forms in the UG. This average score is called the student’s universe score, which is the analogue of true score in CTT. The variance of such universe scores, called universe score variance, can be estimated using the analysis of variance “machinery” employed by G theory.G theory can accommodate numerous designs to examine the measurement characteristics of many kinds of student assessments. If medical educators wish to investigate assessment items as a single source of measurement error on a test, this is a single facet design. There are two types of single-facet designs. If the *same* sample of questions is administered to a cohort of students, we say the design is crossed in that all students (s) respond to all items (i). This crossed design is symbolised as s × i, and read students are crossed within items. If each student takes a different set of items, we have a nested design, which is symbolised i:s meaning that items are nested within students.In most realistic circumstances there are facets in addition to items. Imagine a case-based assessment with four cases and a total of 40 items designed to measure the ability of students about dermatology. In this example, all students take all items; hence, students are crossed within items (s × i), but items are distributed into cases (e.g., 10 items in case 1, 10 items in case 2, 10 items in case 3 and 10 items in case 4). That is, items are nested within cases, and this design is called a two-facet nested design that is symbolised as s × (i:c).The designs discussed in the previous paragraphs are usually called G study designs, and they are associated with the UAO. The principal purpose of such designs is to collect data that can be used to estimate what are called “variance components.” In essence, the set of variance components for the UAO provides a decomposition of the total observed variance into its component parts. These component parts reflect the differential contribution of the various facets; i.e., a relatively large variance component associated with a facet indicates that the facet has a relatively large impact on student marks. For example, in an OSCE, if the variance component for examiners (the examiner facet) is estimated as high, we would conclude that the examiners have not behaved consistently in their rating of the construct of interest.Once variance components are estimated, typically investigators estimate error variances and reliability-like coefficients that are associated with the UG. Such coefficients can range from 0 to 1. One coefficient is called a generalizability coefficient; it incorporates relative error variance. Another coefficient is called a Phi coefficient; it incorporates absolute error variance. Computing these coefficients and error variances requires specifying the D study design which, in turn, specifies the number of conditions of each facet that are (or will be) used in the operational measurement procedure. Relative error variance (and, hence, a generalizability coefficient) is appropriate when interest focuses on the rank ordering of students. Absolute error variance (and, hence, a Phi coefficient) is appropriate when interest focuses on the actual or “absolute” scores of students. Relative error variance (for a so-called “random effects” model) involves all the variance components that are interactions between students and facets. Absolute error variance includes relative error variance plus the variance components for the facets themselves. The square root of these error variances are called standard errors of measurement. They can be used to establish confidence intervals for students’ universe scores. For further information about the these coefficients and error variances, readers may refer to particular books.^2^^,^^3^Knowing the magnitude of estimated variance components enables us to design student assessments that are optimal, at least from a measurement perspective. For example, a relatively small estimated variance component for the interaction of students and items suggests that a relatively small number of items may be sufficient for a test to achieve an acceptable level for a generalizability coefficient.In practice, powerful computer programs are required to estimate variance components, coefficients, and error variances, especially for multifaceted designs. Several G theory software programs have been developed for estimating such statistics (see, for example, http://www.education.uiowa.edu/centers/casma/computer-programs).Variance components can also be estimated using SPSS and SAS, but these packages do not directly estimate coefficients and error variances. The first author is developing an online user friendly application for estimating variance components, for both balanced and unbalanced designs. Using a simple script, readers will be able to print out the estimates of important parameters in G theory. The application is written in R and C^++^ languages and executed by PHP codes. Figure 1 shows a balanced design output from the application.

**Figure 1:**
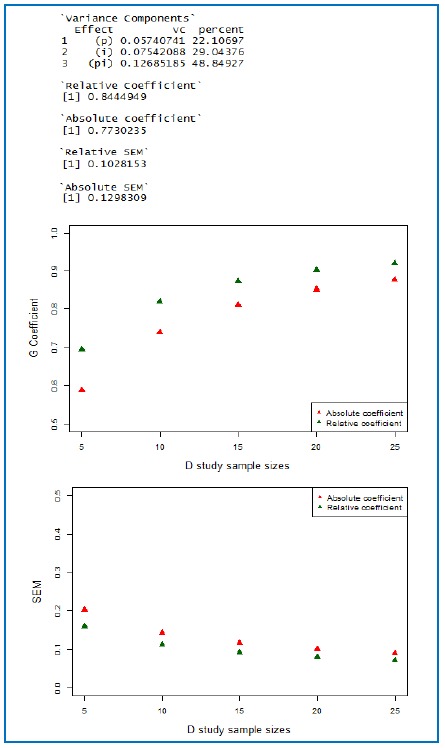
A sample output of G and D studies (p×i); number of items is 12 for the numerical estimates of coefficients and SEMs. The data are taken from Brennan’s book, page 28.

## References

[1] General Medical Council. Workplace based assessment: a guide for implementation. London: General Medical Council; 2012.

[2] Brennan R. Generalizability theory. New York: Springer-Verlag; 2010.

[3] Shavelson R, Webb N. Generalizability theory: a primer. Newbury Park: Sage Publication; 1991.

